# Primary and Secondary Health Impacts of COVID-19 among Minority Individuals in New York State

**DOI:** 10.3390/ijerph18020683

**Published:** 2021-01-14

**Authors:** Lauren A. Clay, Stephanie Rogus

**Affiliations:** 1 Department of Health Administration and Public Health, D’Youville College, Buffalo, NY 14201, USA; 2 School of Global Public Health, New York University, New York, NY 10003, USA; 3 Department of Family and Consumer Sciences, New Mexico State University, Las Cruces, NM 88003, USA; srogus@nmsu.edu

**Keywords:** COVID-19, social determinants of health, health disparities

## Abstract

In addition to the direct health impacts of COVID-19, the pandemic disrupted economic, educational, healthcare, and social systems in the US. This cross-sectional study examined the primary and secondary impacts of the COVID-19 pandemic among low-income and minority groups in New York State using the social determinants of health framework. New Yorkers were recruited to complete a web-based survey through Qualtrics. The survey took place in May and June 2020 and asked respondents about COVID-19 health impacts, risk factors, and concerns. Chi-square analysis examined the health effects experienced by race and ethnicity, and significant results were analyzed in a series of logistic regression models. Results showed disparities in the primary and secondary impacts of COVID-19. The majority of differences were reported between Hispanic and white respondents. The largest differences, in terms of magnitude, were reported between other or multiracial respondents and white respondents. Given the disproportionate burden of COVID-19 on minority populations, improved policies and programs to address impacts on lower-paying essential jobs and service positions could reduce exposure risks and improve safety for minority populations. Future research can identify the long-term health consequences of the pandemic on the social determinants of health among populations most at risk.

## 1. Introduction

The coronavirus disease 2019 (COVID-19) created widespread disruption to the economy, healthcare systems, education, neighborhoods, and social life. Social distancing, quarantine, and travel restrictions led to a reduction in economic activity and job losses, particularly for those in the hospitality, tourism, and transportation sectors [1,2]. Individuals with higher education were more likely to work in jobs compatible with remote work, whereas those with lower education were more likely to work essential jobs [1]. Employment losses were higher among individuals working in jobs requiring face-to-face interaction [1]. Further, food system disruptions resulted in changes in consumer demand, and stockpiling led to concern over food shortages and higher prices [2]. Early evidence suggests that food insecurity dramatically increased as a result of the social and economic impacts of COVID-19 [3,4,5] and health, economic instability, and food insecurity are closely linked [6]. Healthcare workers faced direct infection risks, and high healthcare costs, shortages of protective equipment, and low numbers of ICU beds and ventilators exposed weaknesses in patient care delivery. Access to care for many who may have a higher risk of exposure was limited due to lack of health insurance [2]. Schools across the nation quickly transitioned to online formats, resulting in reductions in employment and productivity for parents, due in part to a loss of childcare, and disruptions in access to free school meals. These shifts called into question the adequacy of student learning in digital environments and impacts on the social lives of students [7,8]. Disaster mental health research suggests that emotional distress is ubiquitous among affected populations and mental health issues, including anxiety and depression, are particularly likely with COVID-19 [9]. The limited literature on the psychological effects of global pandemics has demonstrated increased psychological distress [10], which can result from the primary effects of the disease and secondary effects (i.e., economic depression, loneliness, social isolation) [11]. Studies on the psychological impacts of COVID-19 have found higher reports of loneliness and depression during the pandemic as well as an association between job insecurity and financial concern and symptoms of depression and anxiety [11,12,13].

New York State experienced an early surge in the COVID-19 pandemic in the United States beginning on 8 March 2020 and peaking in positive test daily rates on 14 April 2020, with 11,571 individuals testing positive (43.1% daily positive rate) [14]. Amid challenges with testing capacity and reliability, these numbers are likely an underestimate of the actual disease burden in the state [15,16]. On 20 March 2020, Governor Cuomo issued the Executive Order “New York State on Pause”, a stay-at-home order with closure of all non-essential businesses for the state to slow the spread of the coronavirus [17]. The rising infection and death rates are disproportionately experienced by racial and ethnic minorities, lower-income communities, and essential workers of whom many are from the aforementioned communities. Analysis of infection rates shows that Black and Latino individuals are three times more likely to become infected with COVID-19 and are twice as likely to die than whites in the United States [18,19,20]. Population-level data show that lower-income individuals are less likely to get tested and are more likely to be positive when tested. This corresponds to populations with lower paid jobs that are more likely to be essential such as individuals working in municipal, factory, service, and healthcare settings and therefore at greater risk of exposure [19]. With widespread community disruption and risk factors at the individual, community, and policy levels that create health disparities, the present study takes a social determinants of health approach to examining differential impacts of COVID-19 in New York State [21].

Social determinants of health are “the conditions in the environments where people are born, live, learn, work, play, worship, and age that affect a wide range of health, functioning, and quality-of-life outcomes and risks [22].” Determinants are grouped into five categories for monitoring progress towards improved health as part of the Healthy People 2030 goals in the United States: economic stability that includes housing and nutrition, education, healthcare, neighborhood and built environment, and social and community context [22]. When a disaster or public health emergency occurs, it results in disruption to systems and infrastructure, impacting the routines of daily life and the stability of households and communities [23]. In addition to the primary health impacts of a hazard such as injury, illness, or property damage, there are many secondary health effects that influence the health and well-being of individuals. To more fully understand the health consequences of the pandemic for families, it is important to characterize a broader range of impacts on health determinants. Using the social determinants of health framework [22], this study examines differences in the primary and secondary health impacts of COVID-19 among low-income and minority groups in New York State.

## 2. Materials and Methods

### 2.1. Study Design and Selection of Participants

A cross-sectional web survey asked about COVID-19 health impacts, risk factors, food access, and concerns related to the social determinants of health. The survey was adapted from the validated National Food Access Research Team survey [24,25]. Questions about food access and security were adopted directly from the National Food Access and COVID Research Team (NFACT) survey and validation analysis of the survey showed an alpha value of 0.70 [24]. Additions to the survey included validated questions for anxiety and depression and a set of questions about the COVID-19 impact on employment and health adopted from COVID-19 surveys in the PhenX COVID-19 Toolkit, a battery of consensus measures for “**Phen**otypes and e**X**posures” vetted and assembled by expert working groups funded by the National Human Genome Research Institute [26,27,28,29,30,31,32]. A quota-based non-proportional sample of 415 individuals in New York State, excluding counties in the New York City metropolitan area due to the vastly different context of city and state communities, was recruited by Qualtrics during the phased reopening stages of NY State on Pause [33,34]. Quotas were set to oversample minority (50% Hispanic, 50% African American) individuals with low income or low education (50%) and male respondents (50%) to ensure a sufficient sample size to analyze the experiences of individuals at high risk of adverse COVID-19 risk and consequences. Research shows that in 2018, 89% of white, 88% of Hispanic, and 87% of Black Americans and 81% of Americans with an annual income of less than USD 30,000 used the internet [35]; therefore, a web survey is appropriate for reaching the target population. Participants were recruited from survey panels maintained by Qualtrics [36]. Panel members were eligible to participate if they were aged 18 or older and lived in New York State, excluding New York City. Potential participants were asked about their race, ethnicity, education, income, and gender to target filling the quotas set for the study. Participants that met the inclusion criteria but fell outside of the quotas needed for the sample were ineligible to continue. A total of 1,274 people began the survey, and 475 people were excluded due to ineligibility, such as not living in a recruitment county, not fitting in the quotas, or not consenting to participate. Another 325 participants were removed due to poor quality responses, such as speeding, straight-lining, or providing nonsense responses [37,38]. A response rate is not available with panel data collection through Qualtrics because the research team does not have access to how many people were invited to participate in the survey. Data are only available on how many people started and completed the survey.

### 2.2. Data Collection

Study participants were invited to complete the survey by Qualtrics and reviewed a consent form before starting the survey. Data collection was completed between 14 May and 8 June 2020. The median time for survey completion was 13 minutes. This study was approved by the D’Youville College Institutional Review Board and was exempt as survey data collection was anonymous.

### 2.3. Measures

Race was assessed by asking respondents to select all self-identified races from a list of 13 options. Respondents were coded into race groups of white, Black or African American, other, and more than one race. Hispanic ethnicity was assessed by asking respondents to indicate if they identify as having Hispanic, Latino, or Spanish origins. Respondents were dichotomously categorized as Hispanic or not Hispanic. The variable for race and ethnicity for the present analysis was computed by classifying each respondent as non-Hispanic white, non-Hispanic Black or African American, Hispanic, or other or more than one race (white, Black or African American, Hispanic, other or multiracial).

Direct health impacts of COVID-19 were assessed by asking respondents if they knew anyone who had tested positive, been quarantined, been hospitalized, or died due to the virus and about current mental health. Respondents were classified as having a direct COVID-19 impact if they checked “self” for any of the impact categories and impact on family or friends if they checked “family” or “friend” for any category. Likely depression was assessed by the PHQ-2, a two-item screener for depressive disorders [31]. A cut point of three was used to classify respondents with likely major depressive disorder (83% sensitivity and 90% specificity) [31]. Anxiety was assessed with the GAD-2, a two-item screener for generalized anxiety disorder [30]. A cut point of three was used to classify respondents with likely generalized anxiety disorder (86% sensitivity and 83% specificity) [30].

Secondary health impacts were evaluated using the social determinants of health framework (Table 1). Secondary health impacts include economic stability, education, healthcare, neighborhood and built environment, and social and community contextual factors. Economic stability included measures for income, reduced work and concerns about job security, housing, debt, and food access. Income was assessed by asking participants to select the income range that best described their income in 2019 before taxes (eight categories from less than USD 13,000 to greater than USD 150,000 were categorized as less than USD 25,000, USD 25,000–50,000, and greater than USD 50,000). Reduced work was assessed by asking respondents to check all that apply from a list of job impacts including working more hours, working less hours, furloughed, laid off, working from home, unemployed before the pandemic, or no changes (categorized as reduced work or no reduced work). Concerns about job security, housing, debt, and food security were assessed by asking respondents to indicate on a Likert scale how often (never, sometimes, most of the time, always) they have been concerned about the set of issues. For each issue, responses were categorized as ever (sometimes, most of the time, always) or never (never).

Education was assessed on a Likert scale by asking about how often (never, sometimes, most of the time, always) they were concerned about schooling and responses were categorized as ever (sometimes, most of the time, or always) or never (never). Healthcare was assessed by asking about concern about healthcare access and about health insurance status. Respondents were asked on a Likert scale how often (never, sometimes, most of the time, always) they were concerned about healthcare access since the pandemic began and responses were categorized as ever (sometimes, most of the time or always) or never (never). Health insurance status was assessed by asking respondents if they have public, private, or no health insurance. Health insurance was categorized as insured (public or private insurance) and uninsured (no insurance).

Neighborhood and built environment were assessed on a Likert scale by asking respondents about the frequency (never, sometimes, most of the time, always) of behaviors in the built environment including standing too close to others while getting food, going to bars and restaurants, making reduced grocery shopping trips, and need for more public transportation access during the pandemic. Responses were categorized as ever (sometimes, most of the time, always) or never (never) for each behavior. Access to public transportation was assessed by asking respondents how helpful (not helpful, somewhat helpful, helpful, very helpful) it would be for their household to have more access to public transportation and responses were categorized as helpful (somewhat helpful, helpful, or very helpful) or not helpful (not helpful).

Social and community context includes perceptions of response to the pandemic by different levels of government (city government, state government, federal government, public health such as the Centers for Disease Control and Prevention (CDC)) and communications about protecting households. Respondents were asked to indicate their level of agreement with a set of statements about response to the pandemic (strongly disagree, disagree, somewhat disagree, somewhat agree, agree, strongly agree). Respondents were classified as agreeing the response was effective if they indicated somewhat agree, agree, or strongly agree.

### 2.4. Data Analysis

Sample characteristics and health effects of COVID-19 were described and chi-square analysis was completed to examine differences in health impacts by race and ethnicity. Factors that were statistically significantly different across groups were analyzed in a series of unadjusted logistic regression models (unadjusted odds ratios and 95% confidence intervals reported). Statistical analysis was completed in Stata 16 [39].

## 3. Results

Fifty percent of the respondents were between the ages of 18 and 34 and 55% of the respondents were female. Thirty one percent were Black and 40% were Hispanic. Thirty five percent of the respondents reported an income of below USD 25,000 and 31% reported an educational attainment of high school or less (Table 2).

Chi-square analysis showed significant differences by race and ethnicity for two direct impacts of COVID-19, all six economic stability factors, the single education factor, one healthcare factor, and one neighborhood and built environment factor. There were no significant results for the social and community context factors (Table 3).

The results of the logistic regression analysis revealed disparities in the direct and indirect impacts of COVID-19. The majority of differences were reported between Hispanic and white respondents. The largest differences, in terms of magnitude, were reported between other or multiracial respondents and white respondents (Table 4). A discussion of the direct and indirect impacts follows; indirect impacts are organized according to the social determinants of health.

Direct impacts of COVID-19 were increased among non-white respondents, with 3.5 greater odds of Black or African American respondents knowing a friend or family member infected with COVID-19 compared to white respondents (OR 3.5, 95% CI 1.95, 6.24) (Table 4). The odds of this effect were 3.3 times greater than white respondents for Hispanic respondents (OR 3.3, 95% CI 1.90, 5.75). Hispanic respondents’ odds of having generalized anxiety disorder were two times greater than white respondents’ odds (OR 2.0, 95% CI 1.12, 3.41).

Racial and ethnic differences were observed in measures of economic stability, including income, work disruptions, concerns about job security, paying rent/mortgage, debt, and risk of food insecurity. All groups had greater odds of experiencing at least three of these measures compared to white respondents. Hispanic respondents had increased odds of experiencing all six of the economic stability measures compared to white respondents. Both Black or African American and other or multiracial respondents had greater odds of experiencing three of the six economic stability measures, including concerns about job security and paying rent or mortgage, and risk of food insecurity compared to white respondents. All minority groups had lower odds of greater income in 2019 compared to their white counterparts. Notably, the odds of experiencing concerns about job security (OR 4.0, 95% CI 1.54, 10.44) and paying rent or mortgage (OR 7.9, 95% CI 2.73, 22.84) for other or multiracial respondents were 4.0 and 7.9 times the odds of white participants, respectively.

All racial and ethnic groups experienced increased concerns about schooling compared with white respondents. The odds of experiencing concerns about schooling for Black or African American (OR 2.4, 95% CI 1.32, 4.24), Hispanic (OR 2.8, 95% CI 1.62, 4.95), and other or multiracial (OR 3.5, 95% CI 1.46, 8.52) respondents were more than twice the odds of white respondents.

Hispanic and other or multiracial respondents experienced greater concerns about healthcare access compared to white respondents. The odds of experiencing concerns about healthcare access for Hispanic respondents had double the odds of white respondents (OR 2.0, 95% CI 1.15, 3.45), and other or multiracial respondents had 11.7 times the odds of white respondents (OR 11.7, 95% CI 2.60, 52.28).

Hispanic and other or multiracial respondents were more likely to make behavior changes related to their neighborhood or built environment. Hispanic respondents reported making fewer grocery trips during COVID-19 compared to white respondents (OR 2.3, 95% CI 1.25, 4.32). Other or multiracial respondents had 6.3 greater odds of making fewer grocery trips compared to white respondents (OR 6.3, 95% CI 1.38, 28.37).

## 4. Discussion

This study found differences in the primary and secondary health impacts of COVID-19 among minority groups in New York State. Black or African American and Hispanic respondents were more likely to experience direct impacts of COVID-19 compared to white respondents. Hispanic and Black or African American respondents and respondents identifying as other races or multiracial were more likely to express concern about economic stability and education compared to white respondents. Hispanic and other or multiracial respondents were more likely to express concerns about access to healthcare and make fewer grocery trips during COVID-19.

Plentiful studies highlight the economic and health disparities faced by Black, Indigenous, and people of color (BIPOC). BIPOC are more likely to be impoverished, which places burdens on household budgets that may impact their ability to purchase healthy food, access healthcare, and secure adequate housing [40,41,42]. Low-income individuals are more likely to have low-paying jobs with less flexibility and face challenges meeting all basic needs [42]. In the present sample, 36.6% of the sample reported being essential workers or working outside of the home during stay-at-home orders in NY State. Among essential workers, 16.4% are white, 34.3% are Black or African American, 40.4% are Hispanic, and 8.9% are other or multiracial. In the face of the current research, it is no surprise that respondents of color experienced more concern over their financial circumstances and were more likely to report illness of a family member or friend from COVID-19. Indeed, compared to 42% of all participants reporting knowing anyone diagnosed with the disease in Vermont, this study found a range of 49.3% to 71.1% of low-income, minority participants with a friend or family member with the disease in New York [4].

In light of documented disparities in economic stability, healthcare, and education among BIPOC, this study reported on several concerns indicative of emotional distress experienced by respondents. Other research suggests that the groups most vulnerable to psychological distress due to COVID-19 are those who have contracted the disease, at higher risk to contract the disease, and with pre-existing medical, psychiatric, or substance abuse problems [9]. Confinement to homes can add to emotional distress, and extended confinement, inadequate supplies, difficulty securing medical care/medications, and financial losses can exacerbate emotional distress [9]. Although this study reported higher rates of anxiety among Hispanic New Yorkers and no differences in depression by race/ethnicity, worry among minority individuals over economic stability, healthcare access, and education was observed. Concern over food access and security has been reported elsewhere [4]. Only one study has examined differences in emotional distress due to COVID-19 by income, race, and ethnicity. The study found that low income and unemployment were associated with total stress score, which included worry about the socioeconomic costs of the pandemic [43]. White respondents had the lowest stress scores, Black respondents had intermediate scores, and Asian and Hispanic respondents had the highest scores [43].

This study employed a cross-sectional design; therefore, analyses are not able to characterize causal relationships. To mitigate this limitation, survey questions asked respondents specifically about social determinants of health in relation to COVID-19. The sampling frame was designed to oversample individuals at increased risk of COVID-19. As a result of purposive sampling, generalizations cannot be made about New Yorkers. Given documented COVID-19 disparities, understanding the experiences of individuals with greater risk was privileged over representativeness. The survey was administered through a web-based platform; therefore, individuals without access to the internet were systematically excluded from participation. Given the widespread impact of the pandemic in New York State, the methodology was selected to facilitate timely completion of data collection during phased reopening from NY on Pause and provide an important snapshot of the lived experiences of many New Yorkers.

## 5. Conclusions

Disparities in the direct and indirect impacts of COVID-19 were observed among minority populations in New York State. The majority of differences were reported between Hispanic and white New Yorkers, with the largest differences reported between other or multiracial respondents and white respondents. Given the disproportionate burden of COVID-19 on minority populations, improved policies and programs to address COVID-19 changes to work environments for positions in the service industry, healthcare, and factory settings could reduce risks of exposure and improve worker safety for minority populations. Amid increased risk of COVID-19 infection and death among Hispanic and Black Americans and systemically racist systems in the United States, increased participation of persons of color in pandemic and public health policy making is critical. Pandemic and emergency planning requirements for federal and state agencies could require increased stakeholder participation from diverse groups, and funding for community planning processes would support increased participation by groups that experience a disproportionate burden of adverse outcomes. However, it is important to be mindful of the burden and to also establish policies and processes that support improved outcomes such as racial sensitivity and implicit bias training for all employees in the healthcare and public health sectors and inclusion of measures to identify at-risk groups in hospital and clinic protocols. Future pandemic relief legislation may also consider including additional financial supports for these groups. Future research is needed to understand the long-term health consequences of the pandemic on the social determinants of health among populations most at risk. Research on interventions that best support communities of color is also critical to improve health outcomes.

## Figures and Tables

**Table 1:** Social determinants of health domains aligned with survey measures.

Social Determinant of Health	Measures
Direct COVID-19 impact	Test positive, quarantine, hospitalized, died from COVID-19 for self, family/friends; depression, anxiety
Economic stability	Income, reduced work, concern about job security, concern about housing, concern about debt, concern about food security
Education	Concerns about schooling
Healthcare	Concern about healthcare access, health insurance
Neighborhood and built environment	Standing too close for safety when getting food, going to bars/restaurants, making fewer grocery trips, more access to public transportation
Social and community context	Perceptions of response (city, federal, public health, state government, communications)

**Table 2:** Demographic characteristics of the sample.

Characteristic	Frequency	Percent
**Age**		
18–24	128	30.8
25–34	80	19.3
35–44	75	18.1
45–54	49	11.8
55–64	39	9.4
65+	44	10.6
**Gender**		
Female	230	55.4
Male	180	43.4
Other	5	1.2
**Race/Ethnicity**		
Non-Hispanic white	82	20.6
Black or African American	126	31.6
Hispanic	162	40.6
Other, multirace	29	7.3
**Income**		
<USD 12,999	74	17.8
USD 13,000–24,999	74	17.8
USD 25,000–49,999	106	25.5
USD 50,000–74,999	63	15.2
>USD 75,000	98	23.6
**Employment Before COVID-19**		
Employed, salaried, full-time	98	23.6
Employed, hourly, full-time	81	19.5
Employed, salaried, part-time	29	7.0
Employed, hourly, part-time	40	9.6
Disabled	27	6.5
Retired	41	9.9
Homemaker	19	4.6
Student	32	7.7
Unemployed	48	11.6
**Education**		
Some high school	20	4.8
High school graduate or GED	111	26.8
Some college	97	23.4
Associates degree or technical school	61	14.7
Bachelor’s degree	90	21.7
Postgraduate degree	36	8.7

**Table 3:** Race and ethnic group differences for primary and secondary COVID-19 social and health impacts (chi2).

Row Percentages Reported	Non-Hispanic White		Black or African-American		Hispanic		Other, Multirace	
	***n***	**%**	***n***	**%**	***n***	**%**	***n***	**%**
**Direct Impacts**								
Direct COVID-19 Impact—Self	11	14.3	31	40.3	29	37.7	6	7.8
Direct COVID-19 Impact—Family/Friend ***	32	13.0	87	35.4	110	44.7	17	6.9
Likely Generalized Anxiety Disorder *	26	16.7	44	28.2	77	49.4	9	5.8
Likely Major Depressive Disorder	29	17.5	53	31.9	74	44.6	10	6.0
**Economic Stability**								
Income **								
<USD 25,000	15	10.8	51	36.7	63	45.3	10	7.2
USD 25,000–50,000	19	18.3	34	32.7	38	36.5	13	12.5
>USD 50,000	48	30.8	41	26.3	61	39.1	6	3.9
Reduced work *	21	13.2	48	30.2	77	48.4	13	8.2
Concerns about job security ***	36	14.1	84	32.9	113	44.3	22	8.6
Concerns about paying rent/mortgage ***	31	12.4	81	32.4	114	45.6	24	9.6
Concerns about debt **	41	16.1	78	30.6	116	45.5	20	7.8
Risk of food insecurity since COVID-19 **	23	13.7	53	31.6	78	46.4	14	8.3
**Education**								
Concerns about schooling **	26	12.9	66	32.7	92	45.5	18	8.9
**Healthcare**								
Concerns about healthcare access **	44	16.8	78	29.8	113	43.1	27	10.3
Health insurance	75	21.3	113	32.0	143	40.5	22	6.2
**Neighborhood and Built Environment**								
Standing too close when shopping	46	19.3	74	31.1	99	41.6	19	8.0
Going to restaurants less during COVID-19	49	19.7	72	28.9	108	43.4	20	8.0
Making fewer grocery trips during COVID-19 *	56	17.6	100	31.5	135	42.5	27	8.5
More access to public transit would be helpful	11	11.7	34	36.2	42	44.7	7	7.5
**Social and Community Context**								
City response effective	52	22.1	79	33.6	88	37.5	16	6.8
Communication about COVID-19 effective	59	21.8	92	34.0	101	37.3	19	7.0
Federal response effective	47	24.9	51	27.0	78	41.3	13	6.9
Public health response effective	51	21.2	79	32.8	95	39.4	16	6.6
State response effective	57	21.2	93	34.6	102	37.9	17	6.3

*** *p* < 0.001; ** *p* < 0.01; * *p* < 0.05.

**Table 4:** Odds of primary and secondary COVID-19 impacts by race and ethnicity (unadjusted odds ratios).

COVID-19 Impacts	Non-Hispanic White	Black, African American		Hispanic		Other, Multirace	
		**OR**	**95% CI**	**OR**	**95% CI**	**OR**	**95% CI**
**Direct Impacts**							
Direct COVID-19 Impact—Family/Friend ***	ref	3.5	1.95, 6.24	3.3	1.90, 5.75	2.2	0.93, 5.24
Likely Generalized Anxiety Disorder *	ref	1.2	0.64, 2.09	2.0	1.12, 3.41	1.0	0.39, 2.42
**Economic Stability**							
Income **	ref	0.3	0.17, 0.64	0.4	0.18, 0.67	0.4	0.16, 1.10
Reduced work **	ref	1.8	0.97, 3.30	2.6	1.47, 4.72	2.4	0.98, 5.71
Concerns about job security ***	ref	2.6	1.44, 4.53	3.0	1.70, 5.11	4.0	1.54, 10.44
Concerns about paying rent/mortgage ***	ref	3.0	1.66, 5.27	3.9	2.23, 6.84	7.9	2.73, 22.84
Concerns about debt **	ref	1.6	0.93, 2.85	2.5	1.45, 4.38	2.2	0.91, 5.45
Risk of food insecurity since COVID-19 **	ref	2.4	1.28, 4.48	3.0	1.65, 5.42	3.5	1.33, 9.28
**Education**							
Concerns about schooling **	ref	2.4	1.32, 4.24	2.8	1.62, 4.95	3.5	1.46, 8.52
**Healthcare**							
Concerns about healthcare access ***	ref	1.4	0.80, 2.47	2.0	1.15, 3.45	11.7	2.60, 52.28
**Neighborhood and Built Environment**							
Making fewer grocery trips during COVID-19 *	ref	1.8	0.95, 3.37	2.3	1.25, 4.32	6.3	1.38, 28.37

*** *p* < 0.001; ** *p* < 0.01; * *p* < 0.05.

## Data Availability

The data presented in this study are available on request from the corresponding author. The data are not publicly available at this time. Data will be published upon completion of primary study aims.
